# An Improved Tobacco Mosaic Virus (TMV)-Conjugated Multiantigen Subunit Vaccine Against Respiratory Tularemia

**DOI:** 10.3389/fmicb.2018.01195

**Published:** 2018-06-05

**Authors:** Ahd A. Mansour, Sukalyani Banik, Ragavan V. Suresh, Hardeep Kaur, Meenakshi Malik, Alison A. McCormick, Chandra S. Bakshi

**Affiliations:** ^1^Department of Microbiology and Immunology, New York Medical College, Valhalla, NY, United States; ^2^College of Pharmacy, Touro University California, Vallejo, CA, United States; ^3^Department of Basic and Clinical Sciences, School of Arts and Sciences, Albany College of Pharmacy and Health Sciences, Albany, NY, United States

**Keywords:** *Francisella*, tularemia, subunit vaccine, tobacco mosaic virus, mouse model, intranasal

## Abstract

*Francisella tularensis*, the causative agent of the fatal human disease known as tularemia is classified as a Category A Select Agent by the Centers for Disease Control. No licensed vaccine is currently available for prevention of tularemia in the United States. Previously, we published that a tri-antigen tobacco mosaic virus (TMV) vaccine confers 50% protection in immunized mice against respiratory tularemia caused by *F. tularensis*. In this study, we refined the TMV-vaccine formulation to improve the level of protection in immunized C57BL/6 mice against respiratory tularemia. We developed a tetra-antigen vaccine by conjugating OmpA, DnaK, Tul4, and SucB proteins of *Francisella* to TMV. CpG was also included in the vaccine formulation as an adjuvant. Primary intranasal (i.n.) immunization followed by two booster immunizations with the tetra-antigen TMV vaccine protected 100% mice against i.n. 10LD_100_ challenges dose of *F. tularensis* live vaccine strain (LVS). Mice receiving three immunization doses of tetra-antigen TMV vaccine showed only transient body weight loss, cleared the infection rapidly, and showed minimal histopathological lesions in lungs, liver, and spleen following a lethal respiratory challenge with *F. tularensis* LVS. Mice immunized with the tetra-antigen TMV vaccine also induced strong *ex vivo* recall responses and were protected against a lethal challenge as late as 163 days post-primary immunization. Three immunization with the tetra-antigen TMV vaccine also induced a stronger humoral immune response predominated by IgG1, IgG2b, and IgG2c antibodies than mice receiving only a single or two immunizations. Remarkably, a single dose protected 40% of mice, while two doses protected 80% of mice from lethal pathogen challenge. Immunization of Interferon-gamma (IFN-γ)-deficient mice with the tetra-antigen TMV vaccine demonstrated an absolute requirement of IFN-γ for the generation of protective immune response against a lethal respiratory challenge with *F. tularensis* LVS. Collectively, this study further demonstrates the feasibility of TMV as an efficient platform for the delivery of multiple *F. tularensis* antigens and that tetra-antigen TMV vaccine formulation provides complete protection, and induces long-lasting protective and memory immune responses against respiratory tularemia caused by *F. tularensis* LVS.

## Introduction

*Francisella tularensis* causes the highly lethal disease known as tularemia in humans especially when the infection is acquired through the respiratory route. Tularemia remains endemic in many regions of the world and outbreaks continue to affect thousands each year (Centers for Disease Control and Prevention [CDC], 2002; Tarnvik et al., 2004; Eisen et al., 2008; Centers for Disease Control and Prevention [CDC], 2013; Gurcan, 2014). The pathogenic strains of *Francisella* belong to subspecies *tularensis* (Type A) or subspecies *holarctica* (Type B). Pneumonic tularemia causes 30–60% mortality in untreated cases following inhalation of as few as 10 bacteria (Feldman et al., 2001; Sjostedt, 2007). In addition, *F. tularensis* is categorized among the most prominent threats for use as a bioterror agent as it can be rapidly disseminated by aerosols and can cause widespread severe illness and death (Bossi and Bricaire, 2003; Cronquist, 2004). As *F. tularensis* has been used in bioweapon programs in the past and may potentially be used as a bioterror agent, it poses a clear and present threat to public health. As a result, *F. tularensis* has been classified as Tier 1 Category A Select Agent by the Centers for Disease Control. Antibiotic resistance, either through engineering or natural occurrence, undermines all currently available therapeutic options for treatment of respiratory tularemia. Currently, no FDA approved vaccine is available in the United States for prevention of tularemia. Thus, there is an urgent need for development of safe and effective vaccines.

*Francisella tularensis* is an intracellular pathogen; however, an extracellular phase for *F. tularensis* has also been reported (Forestal et al., 2007; Yu et al., 2008). Interferon-gamma (IFN-γ), tumor-necrosis factor, neutrophils, and other phagocytic cells play important roles in protection against *F. tularensis* infection, although they may be most critical during primary infections (Sjostedt et al., 1996; Chong and Celli, 2010; Allen, 2013). Earlier studies reported that cell-mediated but not the humoral immunity is critical for protection against tularemia, since vaccination of humans with live attenuated but not killed organisms resulted in protection against a virulent strain (Tarnvik, 1989). Nevertheless, protection of mice against *F. tularensis* LVS can in fact be passively transferred to naive animals with immune serum (Kirimanjeswara et al., 2007). Antibodies alone have not to-date successfully protected mice against a challenge with a virulent Type A strain; however; they do slow the course of a Type A infection (Fulop et al., 2001; Conlan et al., 2002). It has also been reported that antibodies can provide therapeutic and prophylactic protection against pulmonary tularemia only in the presence of an active cell-mediated immune (CMI) response (Kirimanjeswara et al., 2007, 2008). Both the CD4 or CD8 cells are required for control of primary *F. tularensis* infection or vaccine induced protective immunity (Wu et al., 2005; Bakshi et al., 2008). These studies demonstrated that generation of both humoral and cellular immunity is required for protection against tularemia.

Majority of tularemia vaccine studies are in pre-clinical stages and no lead vaccine candidate has been identified to-date for evaluation in humans. Multiple approaches for the development of tularemia vaccine have been attempted with little success. Immunization with a killed vaccine caused local reactions, induced poor CMI responses, and failed to provide protection against respiratory *F. tularensis* SchuS4 challenge (Burke, 1977; Baron et al., 2007). The live-attenuated strains including the LVS has succeeded in providing protection against a low dose of *F. tularensis* Schus4 challenge in mice and non-human primates, but they do not offer a high degree of protection against high dose aerosol challenge with *F. tularensis* SchuS4 (Belyi et al., 1995; Oyston and Quarry, 2005; Bakshi et al., 2008; Barry et al., 2009; Mahawar et al., 2013; Marohn and Barry, 2013; Chu et al., 2014). Moreover, reversion to fully virulent form is always a possibility with live-attenuated vaccines. Studies involving subunit vaccine development against tularemia have identified a number of *F. tularensis* antigens which have the capability of inducing a protective immune response (Holm et al., 1980; Eyles et al., 2007). On the other hand, single antigenic components of *F. tularensis* such as GroEL, DnaK, FopA, KatG, LPS O antigens, or Tul4 are safe when used as vaccines, but elicit a limited protective immune response against respiratory tularemia (Conlan et al., 2002; Apicella et al., 2010; Hickey et al., 2011; Kaur et al., 2012). Complex subunit vaccine formulations consisting of more than one purified antigenic component or a mixture of antigens either in the form of outer membrane proteins or *F. tularensis* LVS and SchuS4 lysates have shown improved protection against respiratory challenge with *F. tularensis* (Huntley et al., 2008; Ashtekar et al., 2012; Richard et al., 2014, 2017). However, non-availability of a suitable platform for delivering protective antigens simultaneously through a mucosal route has been the biggest hurdle in development of tularemia subunit vaccine.

In our previous study, we employed a novel approach for the development of a multi-antigen subunit vaccine against tularemia by using TMV-based delivery platform. We have shown that tri-antigen TMV vaccine consisting of OmpA, DnaK, and Tul4 proteins of *F. tularensis* SchuS4 generated a T_H2_ predominated humoral immune response indicated by higher levels of *F. tularensis-*specific IgG1 antibodies and provided only 50% protection in immunized C57BL/6 mice (Banik et al., 2015). In the present study, we refined the TMV-vaccine formulation to improve the level of protection in immunized C57BL/6 mice against respiratory tularemia. We improved the tri-antigen TMV vaccine (OmpA, DnaK, and Tul4) by including dihydrolipoamide succinyl transferase (SucB) protein of *F. tularensis* SchuS4 into a tetra-antigen formulation. A CpG adjuvant was also incorporated into the vaccine formulation to induce a T_H1_ biased humoral immune response. The results from this study demonstrate that tetra-antigen TMV vaccine formulation with CpG adjuvant provides 100% protection in immunized C57BL/6 mice and induces a long-term protection against respiratory tularemia caused by *F. tularensis* LVS.

## Materials and Methods

### Bacterial Strains

*Francisella tularensis* LVS (American Type Culture, ATCC 29684; Rockville, MD, United States) and *F. tularensis* SchuS4 strains used in this study were obtained from BEI Resources, Manassas, VA, United States. All *F. tularensis* LVS strains were handled in a Bio Safety Level-2 (BSL-2) laboratory. The *F. tularensis* SchuS4 strain was handled in a CDC approved BSL-3 facility at New York Medical College. *F. tularensis* LVS and SchuS4 were cultured on MH chocolate agar plates (BD Biosciences, San Jose, CA, United States) and grown for 48 h at 37°C in an atmosphere of 5% CO_2_. Briefly, grown single colonies were picked and resuspended in MH-broth (MHB; BD Biosciences, San Jose, CA, United States) supplemented with 0.021% w/v anhydrous calcium chloride; 0.000138% w/v hydrous magnesium chloride; 0.00021% w/v 10% glucose, 10% v/v; 2.5% ferric pyrophosphate, and 2.5% v/v Isovitalex (BD Biosciences, San Jose, CA, United States). The suspension was incubated at 37°C with shaking at 160 rpm until an OD_600_ of 0.2 was achieved. The active mid-log phase bacteria were aliquoted into sterile 1.5 ml cryovials and stored at -80°C for further use. For challenge experiments, the frozen cultures of *F. tularensis* LVS were streaked on MH-chocolate agar plates and incubated at 37°C in the presence of 5% CO_2_ for 36 h. A bacterial suspension was made in sterile PBS by adjusting OD_600_ to 0.2, which corresponds to 1 × 10^9^ CFU/ml. The bacteria were diluted to achieve a final concentration of 1 × 10^5^ CFU/20 μl and were used in all challenge experiments.

### Mice

All mice experiments were performed according to the guidelines and protocols approved by the IACUC at New York Medical College. For vaccination and challenge studies, wild-type C57BL/6 mice were purchased from Charles River Laboratories. The C57BL/6 IFN-γ knockout (B6.IFN-γ^-/-^) (Dalton et al., 1993) and their corresponding wild-type C57BL/6J (B6.IFN-γ^+/+^) mice were purchased from The Jackson Laboratories. Six-to-eight week old mice of either sex were used in all the vaccination and challenge studies. All mice were maintained in an environmentally controlled and pathogen-free animal facility of the New York Medical College.

### Tobacco Mosaic Virus (TMV)

Tobacco mosaic virus was engineered to express coat protein containing a surface exposed lysine (Smith et al., 2007). Infectious TMV RNA was inoculated onto a 30-day-old *Nicotiana benthamiana* plant, and harvested for virus 10 days later according to previously described protocols (Smith et al., 2006). Briefly, the plant tissue was homogenized in 0.86 M sodium chloride, 0.04% w/v sodium metabisulfite (0.5 g of tissue/ml of buffer), adjusted to a pH of 5.0, heated to 47°C for 5 min, and then chilled to 4°C. Homogenate was centrifuged at 6000 × *g* for 20 min, and then the clarified supernatant was precipitated with 5% poly ethylene glycol (PEG) 8000 at 4°C, and spun at 12,000 × *g* for 10 min at 4°C to recover the virus. PEG pellets were re-suspended in PBS, and re-precipitated with PEG a second time. The final PEG pellets were re-suspended in PBS at 1:10 homogenization volume, and final protein concentration was measured using bicinchoninic acid (BCA) kit.

### Adjuvant

A synthetic endotoxin-free oligodeoxynucleotide comprising of unmethylated CpG motifs (CpG 1826, Invivogen) was blended in the vaccine formulation and 20 μg of CpG was administered with each immunization.

### Expression, Purification, and Conjugation of Recombinant Proteins

The selection and prioritization of *F. tularensis* protein antigens used in the TMV-vaccine formulation was based on the immunoproteomic analysis from previously published studies. Antibodies against OmpA (FTT0831c), DnaK (FTT1269c), Tul4 (FTT0901), and dihydrolipoamide succinyl transferase (SucB) (FTT0077) proteins of *F. tularensis* SchuS4 have been identified exclusively in serum isolated from human patients with a prior history of infection with *F. tularensis* Type A strains, or those vaccinated with *F. tularensis* LVS (Havlasova et al., 2002, 2005; Eyles et al., 2007; Janovska et al., 2007). This was the basis for inclusion of these proteins in our vaccine formulation. For expression of these proteins, genes of *F. tularensis* SchuS4 encoding for OmpA (FTT0831c), DnaK (FTT1269c), Tul4 (FTT0901), and SucB (FTT0077) were cloned in *Escherichia coli* expression vectors, expressed, and purified following our previously published protocols (Banik et al., 2015). The purity of the recombinant proteins was confirmed by SDS-PAGE and western blot analysis using anti-6His antibodies. Each of the recombinant *F. tularensis* SchuS4 protein was chemically conjugated to the surface of TMV. TMV-DnaK, TMV-OmpA, TMV-SucB, and TMV-Tul4 conjugates were generated as described previously with slight modifications (Mallajosyula et al., 2014; Banik et al., 2015; Arnaboldi et al., 2016). The purified TMV 1295.10 expressing a surface lysine (Smith et al., 2006) was mixed at a 1:1 mass ratio for DnaK, OmpA, and SucB to avoid steric hindrance due to the higher molecular weights of these proteins (70, 55, and 60 kD, respectively). For conjugation of Tul4, which has a very low molecular weight of 14.5 kD, the TMV and Tul4 were mixed in a 1:1 molar ratio. The conjugation reaction was carried out by adding 100 mM 2-(*N*-morpholino) ethanesulfonic acid and 500 mM NaCl solution at pH 6.0. Subsequently, EDC hydrochloride followed by NHS were added to the reaction mixture. EDC concentrations and the duration of the reactions were optimized to observe that no “free” protein antigen was left after the reaction, which varied for each individual protein. The conjugation reactions were stopped with 1 mM methylamine, and then conjugates were dialyzed against PBS overnight (Slidalyzer, 10 kD MCO). Following dialysis, the concentration of each TMV–protein conjugate was determined using BCA assay (BCA BioRad). SDS-PAGE using 8–16% gel (BioRad) followed by Coomassie blue staining was performed to visualize TMV–protein conjugates. The TMV–protein conjugates were stored at -20°C until used for immunizations.

### Vaccine Formulation

All the four (DnaK, OmpA, Tul4, and SucB) recombinant protein–TMV conjugates (consisting of 10 μg TMV + 10 μg of each recombinant protein) were mixed at an equal concentration to yield a tetra-antigen TMV vaccine. A group of 10–12 mice each were immunized with tetra-antigen TMV vaccine. Each mouse was inoculated i.n. with 80 μg of tetra-antigen–TMV vaccine, which contained 40 μg of four recombinant *F. tularensis* proteins (10 μg each) and 40 μg TMV with 20 μg of CpG adjuvant (CpG 1826, Invivogen) in 40 μl PBS (20 μl/nare). Control mice received 40 μg TMV (unconjugated) with 20 μg of CpG adjuvant in 40 μl PBS i.n. (20 μl/nare).

### Immunization Schedule

C57BL/6 mice were deeply anesthetized by intraperitoneal (i.p.) injection of a cocktail of ketamine and xylazine to overcome the regurgitation reflexes and to facilitate delivery of the vaccine into the lungs. Groups of mice (*n* = 7–12) were immunized by i.n. route with 40 μl (20 μl/ nostril) volume of tetra-antigen TMV vaccine along with CpG adjuvant in PBS. All mice immunized with tetra-antigen TMV vaccine either did not receive any booster immunization, received an additional booster immunization with a similar dose on day 14, or received two booster immunizations on days 14 and 28 post-primary immunization. The control mice received TMV + CpG in a volume of 40 μl during primary as well as booster immunizations.

### Challenge Studies

All immunized mice were challenged with 1 × 10^5^ CFU (10LD_100_) of *F. tularensis* LVS i.n. on day 49 post-primary immunization. In another experiment, mice were challenged with 10LD_100_ of *F. tularensis* LVS on day 163 post-primary immunization. All mice were deeply anesthetized prior to challenge as described earlier. All mice were weighed daily after the challenge to monitor morbidity and the progression of infection. Mice losing more than 25% body weight were observed closely and if they became immobile were considered moribund and euthanized. Mice were observed for survival for a period of 21 days. All surviving mice were euthanized at the end of the experiment on day 35 post-challenge. The undiluted homogenates of lung, liver, and spleens from these mice were plated on MH chocolate plates. This was done to determine the presence of any residual infection and to establish if immunization with tetra-antigen TMV-vaccine provides a sterilizing immunity as plating whole organ homogenates would detect even a single bacterium in organs of the challenged mice.

To determine the kinetics of bacterial clearance following challenge, the control and immunized mice were challenged with 10LD_100_ of *F. tularensis* LVS and groups of mice (*n* = 3–4) were sacrificed on days 7, 14, and 21 post-challenge. The lungs, livers, and spleens were collected aseptically and homogenized in a bead beater using sterile zirconia beads. The organ homogenates were diluted 10-fold in sterile PBS and plated on MH-Chocolate agar plates to enumerate the bacterial numbers. The limit of detection of bacteria by this assay is 1 × 10^2^ CFU/ml. The plates were incubated for 48 h in the presence of 5% CO_2_ and colonies were counted. Small portions of lungs, livers, and spleens were fixed in 10% formalin, embedded in paraffin, and sectioned and stained with Hematoxylin and Eosin (H&E) stain. The sections were observed and images were taken on a Nikon Eclipse microscope using VIS-Elements AR Version 5.02 software at 10× (Numerical Aperture: 10× 0.30) and 40× (Numerical Aperture: 40× 0.60) magnifications. Number of granulomas were quantitated in uninfected and unvaccinated controls and vaccinated mice on day 7 post-challenge by counting the number of granulomas in 10 random fields under 10× magnification. Liver sections prepared from three mice per group were used for counting granulomas by two independent investigators in a blinded fashion. The data were analyzed by unpaired *t*-test and the *P-*values were determined.

### Antibody Measurements

For determination of antibody levels in immunized mice following immunization and challenge, blood was drawn by retro-orbital venipuncture of anesthetized mice on days 28, 49, 84, or 163 post-primary immunizations, or on day 49 post-challenge and the serum was collected. Control mice were also bled in a similar fashion. All sera samples were stored at -20°C until evaluated for antibody levels. ELISA was performed using lysates made from *F. tularensis* LVS or with OmpA, DnaK, Tul4, or SucB recombinant proteins as previously described (Banik et al., 2015). Briefly, 96-well microtiter plates were coated with 7 × 10^7^ CFU/ml of *F. tularensis* LVS in bicarbonate buffer. *F. tularensis*-specific IgG, IgG1, IgG2b, and IgG2c antibody levels were determined in serum collected from immunized mice on days 28, 49, 84, and 163 post-primary immunization or on day 49 post-challenge. In order to determine the level of antibodies induced against each individual protein of the tetra-antigen TMV vaccine, ELISA plates were coated with purified recombinant OmpA (10 μg/ml), DnaK, Tul4, or SucB (1 μg/ml) proteins. Total IgG antibodies (Invitrogen) were used at 1:10,000 dilutions; while IgG1 (Life Technologies), IgG2b (BioLegend), and IgG2c (Abcam) isotype-specific antibodies were used at 1:1000, 1:500, and 1:500 dilutions, respectively. The antigen-specific total IgG levels were determined in pooled serum from vaccinated mice collected on days 28 and 49 post-primary immunization, or on day 49 post-challenge. All antibody end-point titers were calculated as the inverse of the serum dilution that showed an OD_450_ value 2.5 times above the controls, and expressed as Log_10_ values (Banik et al., 2015).

### *Ex Vivo* Assays

Splenocytes were harvested on day 84 post-primary immunization from a separate group of mice receiving three immunizations with tetra-antigen TMV vaccine or from age-matched control mice. Bone marrow-derived macrophages (BMDMs) derived from C57BL/6 mice were seeded in a 24-well plate and incubated for 24 h at 37°C in 5% CO_2_. BMDMs were infected either with *F. tularensis* LVS or *F. tularensis* SchuS4 at a multiplicity of infection (MOI) of 100, incubated for 2 h at 37°C in 5% CO_2_, and treated with gentamicin (250 μg/ml) for 2 h to kill all extracellular and adherent bacteria. The infected BMDMs were overlaid with splenocytes isolated from immunized or control mice (*n* = 3) at a ratio of 1:1. The co-cultures were allowed to progress for 120 h. The cell culture supernatants were collected at 24, 48, and 120 h and the levels of IL-17 and IFN-γ were determined by Cytometric Bead Array (CBA). The limits of detection for IL-17 and IFN-γ by CBA are 18.9 and 3.7 pg/ml, respectively.

### Statistical Analysis

All results were statistically analyzed using Graphpad Prism Software 6.0 and expressed as mean ± SD. Statistical comparisons were made using one-way ANOVA, unpaired or paired *t*-test. The survivals data were expressed as Kaplan–Meier survival curves and statistical significance was determined by log-rank test. *P* < 0.05 was considered as statistically significant.

## Results

### Tetra-Antigen TMV Vaccine Formulation

**FIGURE 1:**
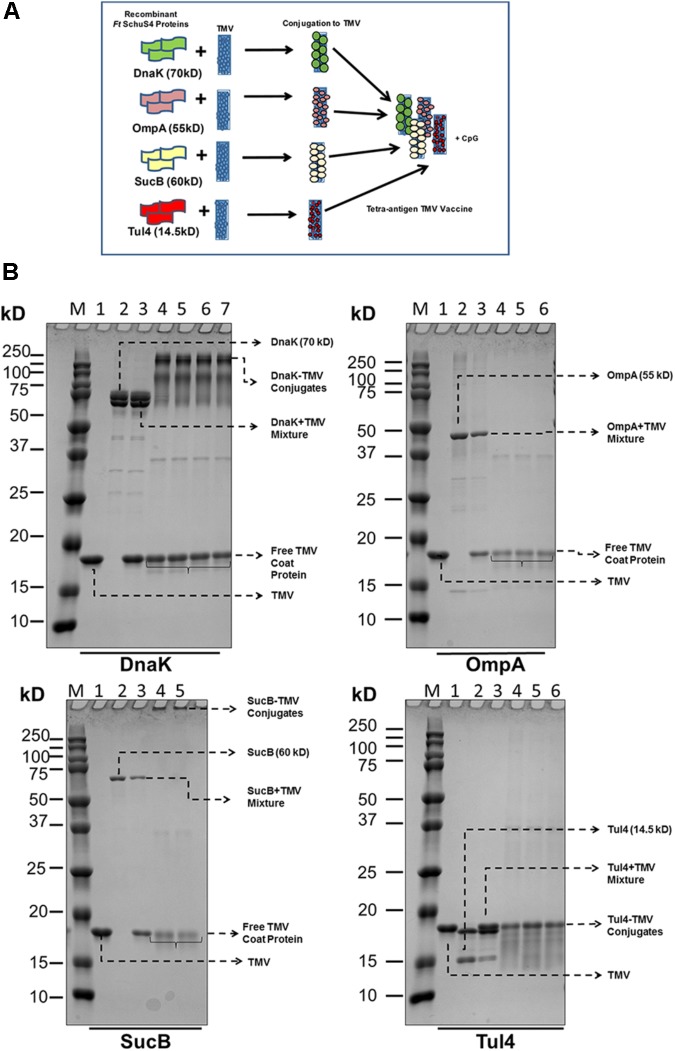
Design and formulation of the tetra-antigen TMV vaccine. **(A)** To the tri-antigen TMV vaccine consisting of OmpA, DnaK, and Tul4, a fourth protein antigen, dihydrolipoamide succinyl transferase (SucB), was added to prepare a tetra-antigen TMV vaccine. Each recombinant protein antigen was conjugated to TMV separately and then mixed in equal concentrations to generate the tetra-antigen vaccine. The CpG 1826 adjuvant was also included in the vaccine formulation. **(B)** Conjugation of *F. tularensis* SchuS4 recombinant proteins to TMV. Recombinant proteins purified by Nickel affinity chromatography were conjugated to TMV as described in the section “Materials and Methods.” The protein–TMV conjugation was visualized by the disappearance of free antigen protein and the appearance of higher molecular weight complexes. M, protein molecular weight marker; 1, TMV; 2, recombinant protein; 3, protein+TMV mixture; 4–7, protein–TMV conjugate replicates from several conjugation reactions.

The design of tetra-antigen TMV vaccine formulation is shown in **Figure 1A**. It has been reported earlier that not all coat proteins of TMV interact directly with the subunit proteins, and this interaction is largely governed by steric hindrances caused by the molecular weight of the interacting protein (Mallajosyula et al., 2014). Therefore, not every individual coat protein of the TMV will interact with subunit proteins larger than its molecular weight of 17 kD. As shown in **Figure 1A**, the larger the subunit protein, the fewer molecules of the subunit protein will associate with the TMV due to steric hindrance at the virus surface. A successful conjugation of recombinant DnaK, OmpA, SucB, and Tul4 to TMV was observed by the disappearance of free protein and/or formation of high molecular weight complexes. The optimal conjugation conditions for TMV-DnaK were 3 mM EDC for 3 h; 6 mM EDC for 3 h for TMV-OmpA; 3 mM EDC for 2 h for TMV-SucB; and 4 mM EDC for 2 h for TMV-Tul4. The reproducibility of the conjugation reaction was confirmed by running replicate samples once the conjugation reactions were optimized (**Figure 1B**).

### Immunization With Tetra-Antigen TMV Vaccine Provides 100% Protection Against a Lethal *F. tularensis* LVS Challenge

**FIGURE 2:**
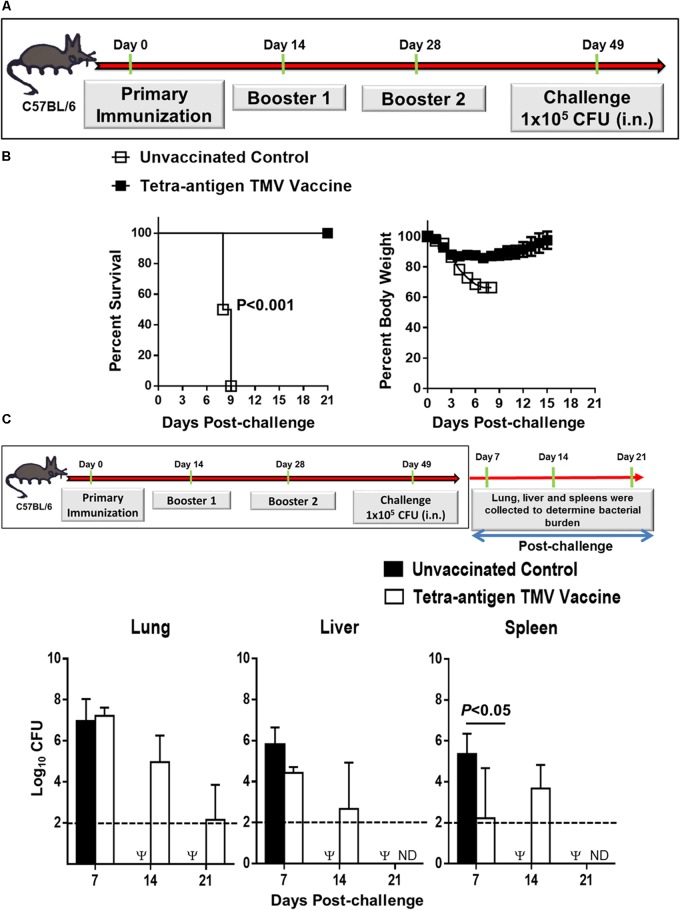
Immunization with tetra-antigen TMV vaccine provides 100% protection and the immunized mice clear infection rapidly following a lethal *F. tularensis* LVS challenge. **(A)** C57BL/6 mice were immunized i.n. on days 0, 14, and 28 with tetra-antigen TMV vaccine formulation as described in the section “Materials and Methods” and challenged i.n. with 1 × 10^5^ CFU (10LD_100_) of *F. tularensis* LVS on day 49 post-primary immunization. **(B)** Mice were observed for mortality and morbidity by monitoring survival and body weights. The survival results are expressed as Kaplan–Meier survival curves and the statistical analysis was performed by log-rank test. The body weight data are represented as mean ± SD (*n* = 10–12 mice/group). The results are representative of two independent experiments conducted. **(C)** Kinetics of bacterial clearance in mice immunized and challenged as shown. The organs were harvested on days 7, 14, and 21 post-challenge and the bacterial burden in the lung, liver, and spleen of vaccinated and control mice was determined. The results represent the means ± standard errors of CFU counts (*n* = 3–4 mice/group/time point) and are from a single experiment conducted. The data were analyzed by one-way ANOVA followed by Bonferroni’s correction and *P-*values were determined. The dotted line represents the limit of detection. Ψ, all control mice succumbed to infection by day 14 post-challenge and hence were not available for comparisons; ND, not detected.

In our previous study, we have demonstrated that immunization with a tri-antigen vaccine consisting of DnaK, OmpA, and Tul4 protected 50% of C57BL/6 mice against an i.n. 10LD_100_ challenge dose of *F. tularensis* LVS. However, to achieve this degree of protection, five immunizations by i.n. and subcutaneous routes were needed (Banik et al., 2015). In this study, we reformulated the tri-antigen TMV vaccine to include an additional antigen SucB, to generate a tetra-antigen TMV vaccine. We also included a CpG adjuvant to our tetra-antigen TMV vaccine to boost a T_H1_ biased immune response (**Figure 1A**). Additionally, the number of immunizations than those used for the tri-antigen vaccine were also reduced. Mice received a primary immunization on day 0 and two booster immunizations on days 14 and 28 post-primary immunization. The immunized mice were challenged i.n. on day 49 post-primary immunization with 10LD_100_ dose of *F. tularensis* LVS (**Figure 2A**). One hundred percent of the vaccinated mice survived the challenge, while 100% of unvaccinated control mice died or were removed from the study due to their moribund state by day 9 post-challenge. The vaccinated mice showed an initial reduction in their body weight for the first 3 days, which remained stable for next 8–9 days post-challenge before starting to regain their pre-challenge body weights. The unvaccinated control mice, however, experienced severe weight loss and either did not survive or were euthanized (**Figure 2B**). These results demonstrate that the tetra-antigen TMV vaccine is fully protective against a lethal respiratory challenge with *F. tularensis* LVS.

We next investigated the kinetics of bacterial clearance in vaccinated mice following challenge. Mice received three immunizations as described above and were challenged on day 49 post-primary immunization. Mice were euthanized on days 7, 14, and 21 post-challenge and bacterial burden was quantitated in their lungs, livers, and spleens. In the lungs of the immunized mice, the bacterial numbers were similar to those observed for the unvaccinated controls on day 7 post-challenge. However, bacterial numbers declined on days 14 and 21 (**Figure 2C**). Bacteria were cleared completely from the lungs of vaccinated mice by day 35 post-challenge as observed by plating the whole organ homogenate on MH-chocolate agar plates that would detect the presence of even a single bacterium in lungs of the challenged mice. These results indicated that the tetra-antigen TMV-vaccine provides a sterilizing immunity (not shown). In livers of the vaccinated mice, the bacterial numbers were not different from that observed for unvaccinated controls on day 7 post-challenge. The spleens of vaccinated mice harbored significantly less number of bacteria than the unvaccinated controls on day 7 post-challenge. Bacterial numbers in livers and spleens declined on day 14 (similar to lung clearance) and were completely cleared from livers and spleen of the immunized mice by day 21 post-challenge. The unvaccinated control mice either succumbed to infection or were moribund and euthanized; hence, were not available for comparisons on days 14 and 21 (**Figure 2C**). These results indicate that immunization with the tetra-antigen TMV vaccine confers a protective immune response capable of providing fully sterilizing immunity.

### Immunization With Tetra-Antigen TMV Vaccine Results in Minimal Pathology in Lung, Liver, and Spleen Following a Lethal Challenge With *F. tularensis* LVS

**FIGURE 3:**
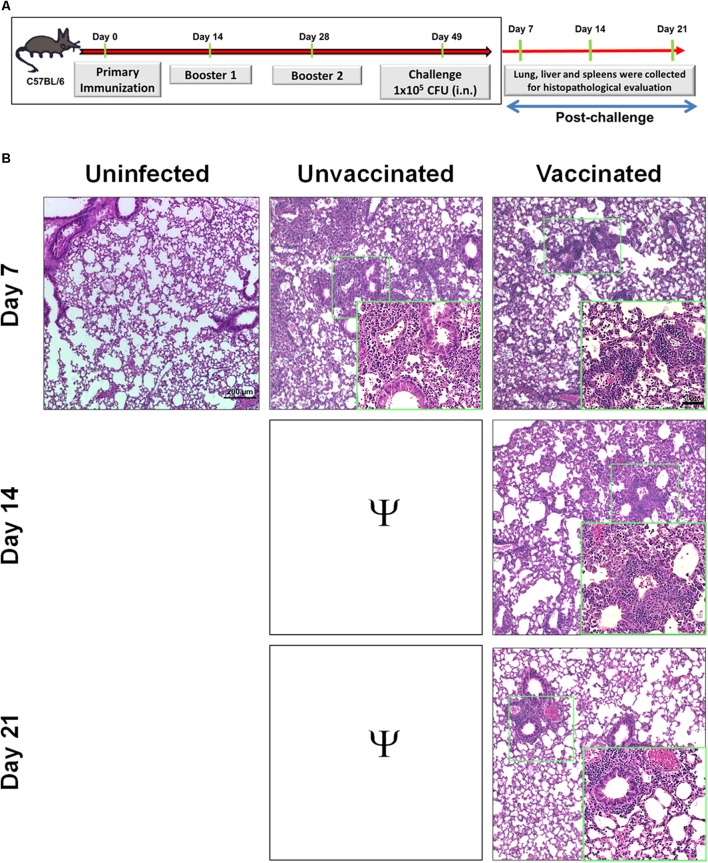
Immunization with tetra-antigen TMV vaccine results in minimal pathology in lung following a lethal challenge with *F. tularensis* LVS. **(A)** C57BL/6 mice were immunized and challenged as shown. Lungs were collected from unvaccinated controls as well as vaccinated mice on days 7, 14, and 21 post-challenge. Hematoxylin and eosin stained sections of lung **(B)** were evaluated for histopathological lesions at the indicated times post-challenge. The experiments were conducted with 3–4 mice/group/time point and the representative images are shown. The sections were observed and images were taken on a Nikon Eclipse microscope using VIS-Elements AR Version 5.02 software. Small green boxes represent the area shown at 40× magnification in insets (magnification 10×; insets 40×; bar in 10× = 200 μM; bar in 40× = 50 μM). Ψ, all unvaccinated mice succumbed to infection by day 14 post-challenge and hence were not available for comparisons.

Since mice vaccinated with tetra-antigen TMV vaccine cleared bacteria from lung, liver, and spleen following *F. tularensis* LVS challenge, it was of interest to examine if bacterial clearance was associated with reduction in the histopathological lesions in these organs as an additional measure of the vaccine’s protective efficacy. Portions of lung, liver, and spleen from mice sacrificed on days 7, 14, and 21 post-challenge (**Figure 3A**) were fixed in 10% formalin, embedded in paraffin, and sectioned. The H&E-stained sections were evaluated for histopathological lesions.

**FIGURE 4:**
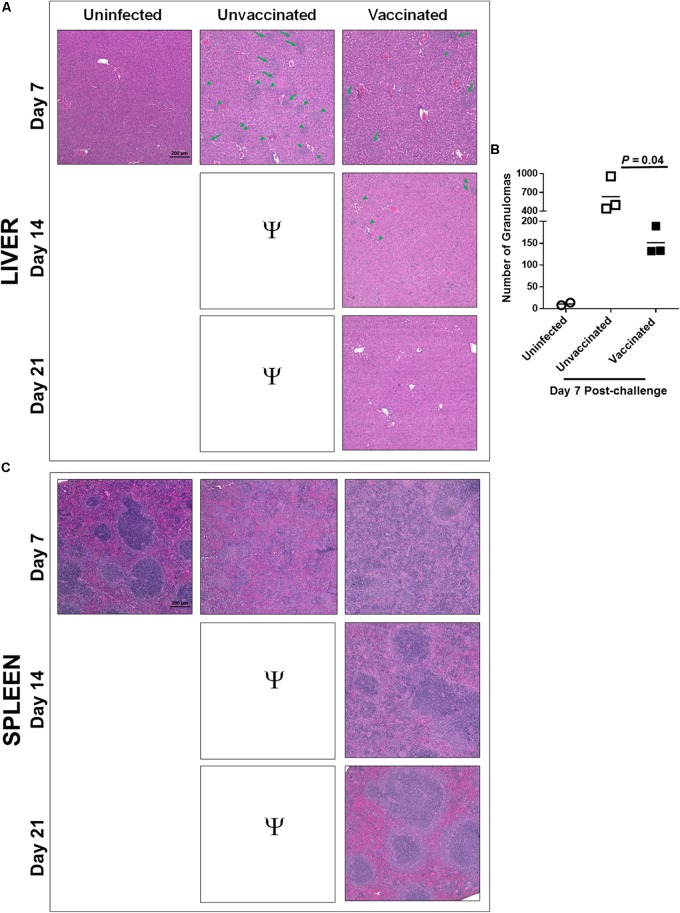
Immunization with tetra-antigen TMV vaccine results in minimal pathology in liver and spleen following a lethal challenge with *F. tularensis* LVS. C57BL/6 mice were immunized and challenged as shown in **Figure 3A**. Liver and spleens were collected from unvaccinated controls as well as vaccinated mice on days 7, 14, and 21 post-challenge. Hematoxylin and eosin-stained sections of liver **(A)** were evaluated for histopathological lesions at the indicated times post-challenge. The arrows and arrow heads indicate granulomas in livers of unvaccinated controls and mice immunized with tetra-antigen TMV vaccine. **(B)** Number of granulomas were quantitated in uninfected and unvaccinated controls on day 7 post-challenge by counting the number of granulomas in 10 random fields under 10× magnification by two independent investigators in a blinded fashion (*n* = 3 mice/group). The data were analyzed by unpaired *t*-test and the *P-*values were determined. **(C)** Hematoxylin and eosin stained sections of spleens were evaluated for histopathological lesions at the indicated times post-challenge. The experiments were conducted with 3–4 mice/group/time point and the representative images are shown. The sections were observed and images were taken on a Nikon Eclipse microscope using VIS-Elements AR Version 5.02 software (magnification 10×; bar = 200 μM). Ψ, all unvaccinated mice succumbed to infection by day 14 post-challenge and hence were not available for comparisons.

The unvaccinated control mice showed severe tissue damage and extensive necrotic lesions in lung, liver, and spleen on day 7 post-challenge. The lungs of immunized mice revealed fewer and smaller inflammatory foci on day 7 post-challenge compared to those observed in lungs of control unvaccinated mice. The number of inflammatory foci decreased and started to resolve by day 14. The normal lung architecture with the exception of few small inflammatory foci was restored by day 21 post-challenge (**Figure 3B**). Livers of the vaccinated mice showed significantly lower number of granulomas compared to those observed in the unvaccinated mice on day 7 post-challenge (**Figures 4A,B**). The granulomas gradually reduced in size by day 14 post-challenge and were no longer observed by day 21 post-challenge in the livers of vaccinated mice (**Figure 4A**). Spleens of unvaccinated mice revealed complete disruption of the splenic architecture, with necrotic splenitis observed on day 7 post-challenge. Similar to lung and liver on day 7 post-challenge, spleen lesions in the vaccinated mice were far less severe and showed only mild disruption of the splenic architecture with greater numbers of infiltrating cells. These lesions started to resolve by day 14 and the normal splenic architecture was restored by day 21 post-challenge (**Figure 4C**). Since all the unvaccinated mice died before day 14, they were not available for comparisons. Collectively, these results demonstrate that mice immunized with tetra-antigen TMV vaccine clear *F. tularensis* infection very efficiently without causing extensive pathological lesions in the lung, liver, or spleen. Furthermore, vaccinated mice showed complete resolution of inflammatory lesions in the lung, liver, and spleen by day 21 post-challenge.

### Tetra-Antigen TMV Vaccine Induces a Potent Humoral Immune Response in Immunized Mice

**FIGURE 5:**
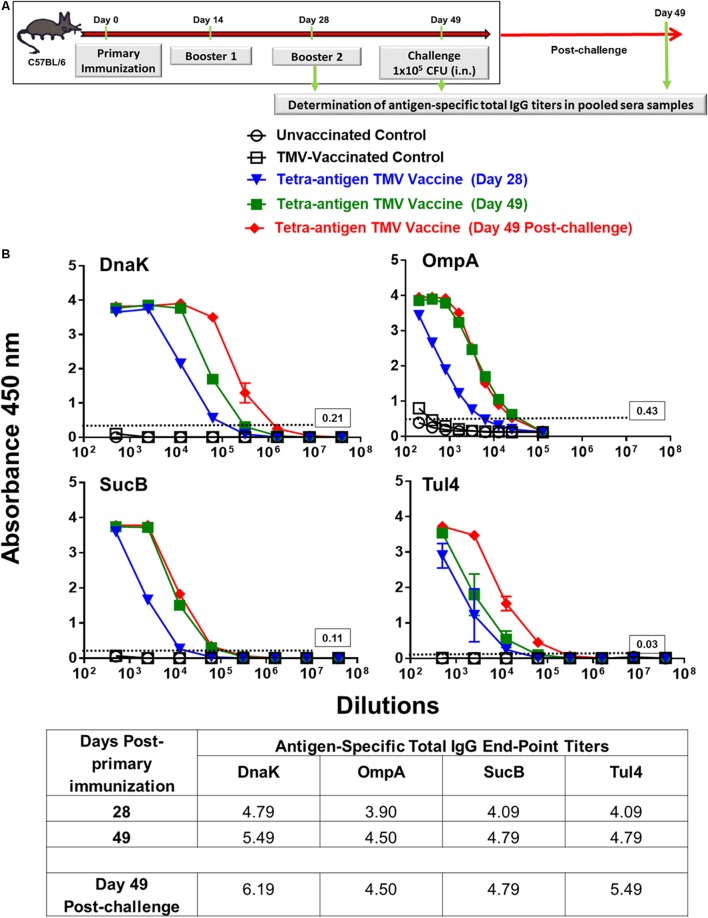
Total IgG antibody responses in immunized mice pre- and post-challenge against each individual protein of tetra-antigen TMV vaccine. **(A)** C57BL/6 mice were vaccinated with tetra-antigen TMV vaccine, TMV alone, or left unvaccinated. Mice were challenged on day 49 post-primary immunization. **(B)** Antigen-specific total IgG antibody titers were determined at the indicated times in pooled serum samples from immunized mice (*n* = 3 mice/group) by ELISA. Each pooled sample was run in duplicate. The results are expressed as absorbance at 450 nm (mean ± standard deviation). The cut-off absorbance values used to determine the end-point titers are indicated by dashed lines and the actual values are shown in the box (top panels). The end-point titers are represented as Log_10_ values (bottom panel).

We determined the contribution of each of the antigen in our vaccine formulation to the generation of antibody responses. Pooled serum samples (*n* = 3 mice) from immunized mice collected on days 28 and 49 post-primary immunization as well as on day 49 post-challenge were used for the determination of total IgG levels against each individual protein (**Figure 5A**). The strongest antibody responses were generated against DnaK followed by Tul4, SucB, and OmpA proteins. An amplification of antibody responses against these proteins was observed on day 49, i.e., after receiving the second booster immunization on day 28 post-primary immunization. These responses were further enhanced when antibody levels were determined on day 49 post-challenge, with DnaK being the most immunogenic followed by Tul4, SucB, and OmpA protein antigens (**Figure 5B**).

We also investigated the humoral immune response in immunized mice receiving none, one, or two booster immunizations with tetra-antigen TMV vaccine. The levels of total IgG, IgG1, IgG2b, and IgG2c were determined using *F. tularensis* LVS lysates. Mice receiving single immunization showed antibody responses predominated by *F. tularensis-*specific IgG1 antibodies. The total IgG as well as the levels of all other antibody isotypes were significantly higher in mice receiving a booster immunization on day 28 post-primary immunization. The antibody levels in mice receiving two booster immunization on days 14 and 28 post-primary immunization were significantly lower than those observed for mice receiving a single booster immunization. As observed for humoral responses against each individual protein in mice immunized with two booster immunizations, the overall total IgG, IgG1, and IgG2c responses were significantly elevated when monitored on day 49 post-challenge compared to those observed on day 49 post-primary immunization (**Table 1**). Collectively, these results demonstrate that the tetra-antigen TMV vaccine is capable of inducing strong antibody-mediated immune responses in immunized mice.

**Table 1:** Antibody responses in mice immunized with tetra-antigen TMV vaccine receiving none, one, or two booster immunizations.

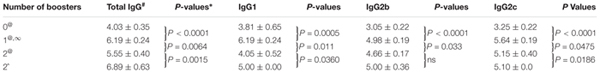


*^#^Log_*10*_ titers expressed as mean ± SD (*n* = 3–4 mice/group). ^@^Antibody levels determined on day 49 post-primary immunization. ^∞^These values are same as shown in **Table 3** and are included in **Table 1** only for comparisons with other immunization regimens. ˆDay 49 post-challenge. ^∗^*P*-values were determined by paired *t*-test. ns = not significant.*

### Tetra-Antigen TMV Vaccine Induces Potent Recall Response in Immunized Mice

**FIGURE 6:**
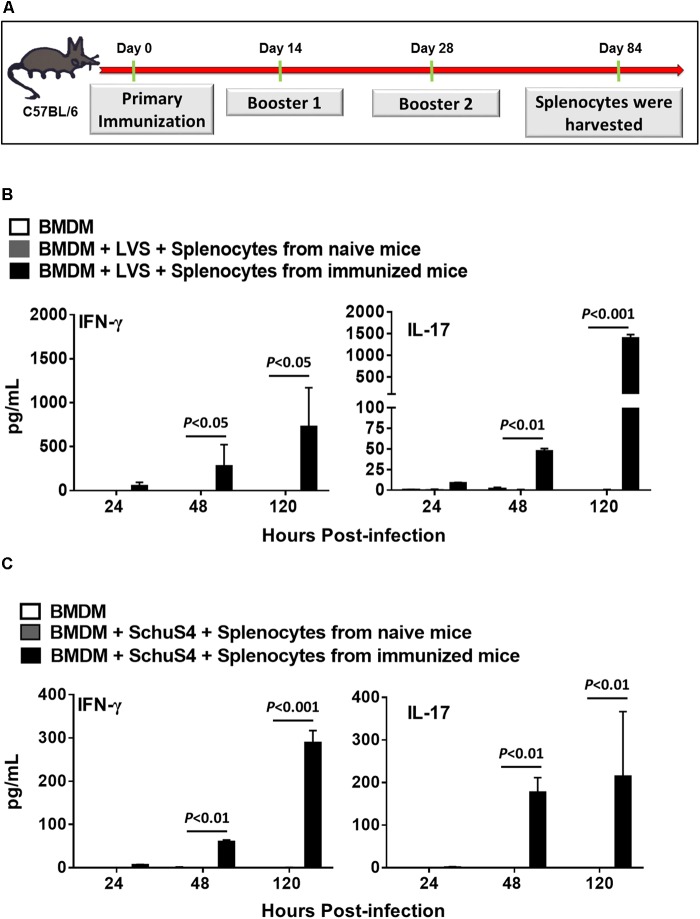
Tetra-antigen TMV vaccine induces potent recall response in immunized mice. **(A)** C57BL/6 mice (*n* = 3–4) were immunized as shown. Mice were sacrificed on day 84 post-primary immunization and single cell spleen suspensions were prepared. BMDMs isolated from syngeneic C57BL/6 mice were infected either with *F. tularensis* LVS **(B)** or *F. tularensis* SchuS4 **(C)** at an MOI of 100 (100:1 bacteria:cell ratio), for 2 h followed by gentamicin treatment for 2 h. The infected BMDMs were then overlaid with splenocytes isolated from immunized mice at 1:1 ratio. Splenocytes isolated from naïve mice (*n* = 3) were used as controls. The culture supernatants from co-culture were analyzed for IFN-γ and IL-17 levels 24, 48, and 120 h later by CBA. The limits of detection for IL-17 and IFN-γ by CBA are 18.9 and 3.7 pg/ml, respectively. The results are representative of two independent experiments. The data were analyzed by one-way ANOVA followed by Bonferroni’s correction and *P-*values were determined.

We determined the duration of protective immune responses induced in mice receiving two booster immunizations with tetra-antigen TMV vaccine by performing *ex vivo* recall response assays. Mice receiving two booster immunizations of tetra-antigen TMV vaccine (**Figure 6A**) were sacrificed on day 84 post-primary immunization and single-cell spleen suspensions were prepared. BMDMs isolated from syngeneic C57BL/6 mice were infected either with *F. tularensis* LVS or *F. tularensis* SchuS4 at an MOI of 100 (100:1 bacteria cell ratio), for 2 h followed by gentamicin treatment for 2 h. The infected BMDMs were then overlaid with splenocytes isolated from immunized mice at 1:1 ratio. Splenocytes isolated from naïve mice were used as controls. The culture supernatants from co-culture were analyzed for IFN-γ and IL-17 levels 24, 48, and 120 h later. It was observed that splenocytes from immunized mice co-cultured with BMDMs infected with *F. tularensis* LVS resulted in significantly higher levels of IFN-γ and IL-17 in culture supernatants collected after 48 and 120 h of co-culture compared to the naïve controls (**Figure 6B**). Similar results were obtained when BMDMs were infected with *F. tularensis* SchuS4 and co-cultured with splenocytes from immunized mice (**Figure 6C**). Collectively, these results demonstrate that the tetra-antigen TMV vaccine is capable of inducing long-lasting recall responses.

### Tetra-Antigen TMV Vaccine Induces Long-Lasting Immunity Against Lethal *F. tularensis* LVS Challenge

**FIGURE 7:**
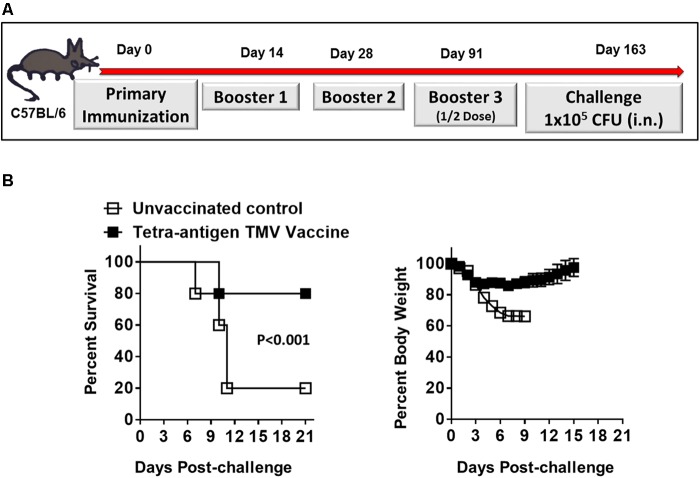
Tetra-antigen TMV vaccine induces long-lasting immunity against lethal *F. tularensis* LVS challenge. **(A)** C57BL/6 mice were immunized i.n. on days 0, 14, and 28 with tetra-antigen TMV vaccine. These mice received an additional booster with half the dose on day 91 post-primary immunization and were challenged i.n. with 1 × 10^5^ CFU (10LD_100_) of *F. tularensis* LVS on day 163 post-primary immunization. **(B)** Mice (*n* = 7/group) were observed for mortality and morbidity by monitoring survival and body weights. The results shown are from a single experiment conducted. The data are represented as mean ± SD. The survival results are expressed as Kaplan–Meier survival curves and the statistical analysis was performed by log-rank test.

We next determined the duration of immunity in mice immunized with tetra-antigen TMV vaccine followed by two booster immunizations on days 14 and 28 post-primary immunization. The immunized mice were bled on day 84 post-immunization to determine the antibody levels. The immunized mice were boosted again on day 91 post-primary immunization using half of the vaccination dose that they received during primary and booster immunizations (**Figure 7A**). The mice were bled, challenged with 10LD_100_ dose of *F. tularensis* LVS i.n. on day 163 post-primary immunization, and observed for morbidity and mortality. It was observed that 80% of immunized mice survived the challenge, and after an initial weight loss, regained their pre-challenge body weights (**Figure 7B**). The antibody levels in these immunized mice on day 84 post-primary immunization were lower across the board (**Table 2**) compared to those observed on day 49 post-primary immunization (**Table 1**). However, these responses, especially for total IgG, IgG1, and IgG2c were significantly higher on day 163 compared to those observed on day 84 (**Table 2**) indicating that the enhanced antibody levels are in response to the additional half dose booster that these mice received on day 91 post-primary immunization. Collectively, these results demonstrate that immunization with tetra-antigen TMV vaccine induces potent memory as well a long-lasting protective immune response in vaccinated mice.

**Table 2:** Antibody responses in mice immunized with tetra-antigen TMV vaccine.

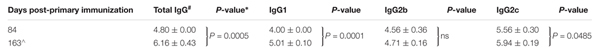


***^#^**Log_*10*_ titers expressed as mean ± SD (*n* = 3–4 mice/group). ˆMice received an additional half dose booster on day 91 post-primary immunization. ^∗^*P*-values were determined by paired *t*-test. ns = not significant.*

#### Protective Efficacy of Tetra-Antigen TMV Vaccine After One and Two Immunizations and Dependence on IFN-γ for the Protection Induced by the Tetra-Antigen TMV Vaccine

**FIGURE 8:**
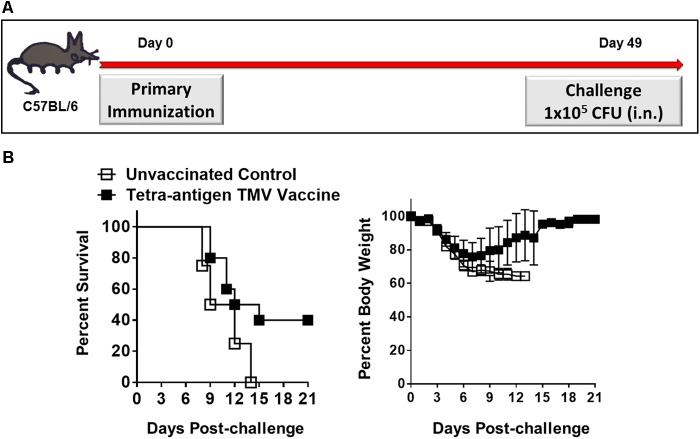
Single dose efficacy of the tetra-antigen TMV vaccine. **(A)** C57BL/6 mice were immunized i.n. on day 0 with tetra-antigen TMV vaccine and challenged i.n. with 1 × 10^5^ CFU (10LD_100_) of *F. tularensis* LVS on day 49 post-primary immunization. **(B)** Mice (*n* = 7/group) were observed for mortality and morbidity by monitoring survival and body weights. The results are representative of two independent experiments conducted. The data are represented as mean ± SD. The survival results are expressed as Kaplan–Meier survival curves and the statistical analysis was performed by log-rank test.

**FIGURE 9:**
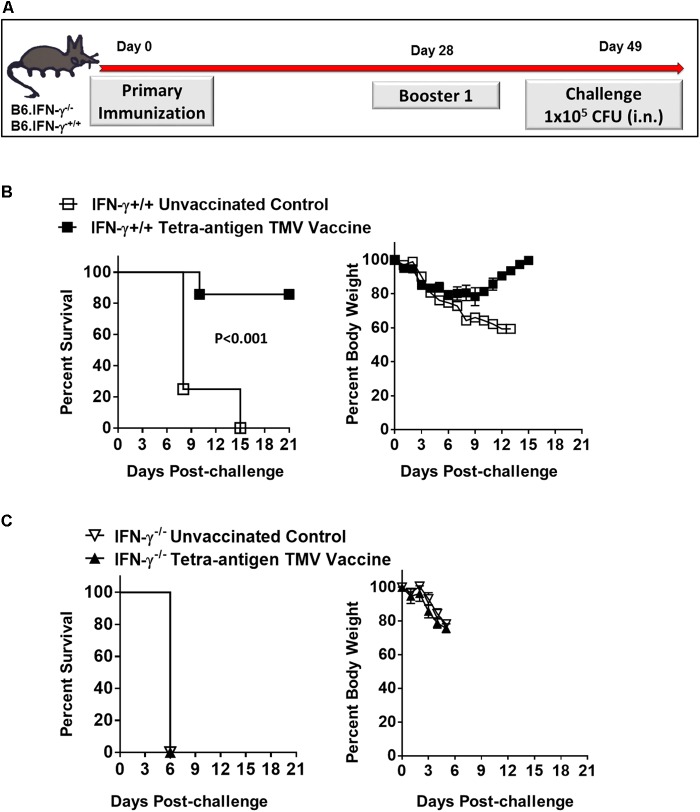
Protective efficacy after one booster immunization, and dependence on IFN-γ for the protection induced by the tetra-antigen TMV vaccine. **(A)** The wild-type (B6.IFN-γ^+/+^) and IFN-γ knockout (B6.IFN-γ^-/-^) mice immunized i.n. on days 0 and 28 with the tetra-antigen TMV vaccine were challenged i.n. with 1 × 10^5^ CFU (10LD_100_) of *F. tularensis* LVS on day 49 post-primary immunization. The B6.IFN-γ^+/+^ **(B)** and B6.IFN-γ^-/-^ **(C)** mice (*n* = 7/group) were observed for mortality and morbidity by monitoring survival and body weights. The data are represented as mean ± SD. The results are representative of two independent experiments for B6.IFN-γ^+/+^ mice, and a single experiment for B6.IFN-γ^-/-^ mice. The survival results are expressed as Kaplan–Meier survival curves and the statistical analysis was performed by log-rank test.

Our preceding results demonstrated that a primary immunization followed by two booster immunizations on days 14 and 28 protected 100% mice against a lethal i.n. challenge with *F. tularensis* LVS. We next investigated if the same protective efficacy can be retained by reducing the number of booster immunizations. C57BL/6 mice either received only one immunization (day 0) or an additional booster immunization on day 28 (**Figures 8A**, **9A**) using similar doses of tetra-adjuvant TMV vaccine as described above. The immunized mice were challenged with 1 × 10^5^ CFU of *F. tularensis* LVS i.n. on day 49 post-primary immunization. As observed earlier, all the unvaccinated control mice succumbed to infection by day 13 post-challenge. Remarkably, 40% of the vaccinated mice that received a single vaccine dose (day 0) survived the challenge. A drastic weight loss was observed in unvaccinated control mice; while in the vaccinated group, all the surviving mice regained their pre-challenge body weights after an initial loss (**Figure 8B**). Survival from lethal i.n. challenge was improved to ∼80% after mice received one booster immunization on day 28. Challenged mice experienced a transient body weight loss for the first 4 days post-challenge, but quickly regained their pre-challenge body weights. One hundred percent of unvaccinated controls died with an associated weight loss as observed earlier (**Figure 9B**). Determination of bacterial burdens in all the surviving mice on day 45 post-challenge resulted in recovery of no bacteria, indicating generation of a sterilizing immunity in the vaccinated mice. In another experiment, C57BL/6 mice were immunized on days 0 and 28 with a mixture of unconjugated recombinant DnaK, OmpA, SucB, and Tul4 proteins (10 μg/each) along with CpG adjuvant (20 μg) using the concentrations similar to those present in the tetra-antigen TMV vaccine. The mice were challenged i.n. with 1 × 10^5^ CFU of *F. tularensis* LVS. It was observed that only 50% mice survived the challenge indicating that the protective efficacy declines if a mixture of unconjugated proteins rather than TMV-conjugated proteins are used in a vaccine formulation (data not shown). Collectively, these results demonstrate that there is potential for further optimization of an effective single dose immunization, or full protection with a single booster immunization. These data demonstrate the greatly improved protective efficacy of the tetra-antigen TMV vaccine that includes TMV-SucB.

**FIGURE 10:**
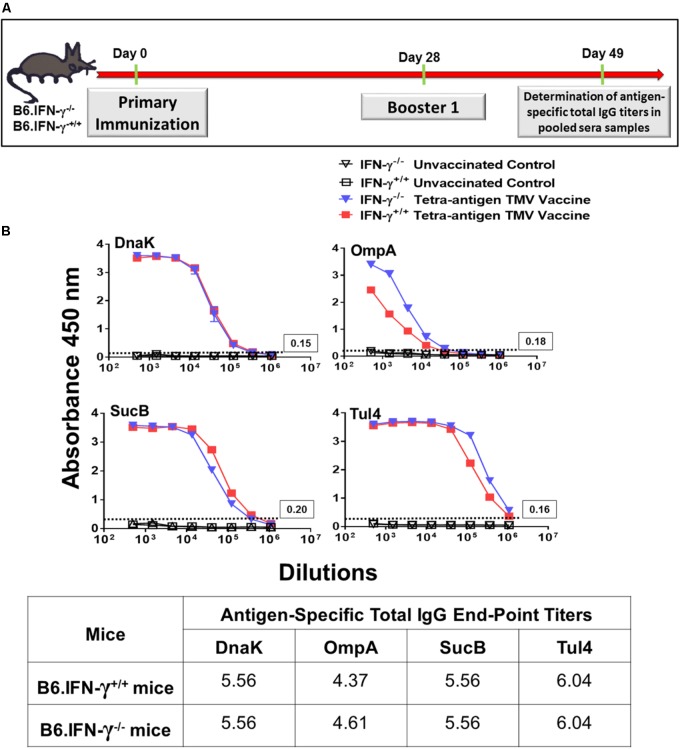
Total IgG antibody responses in wild-type and B6.IFN-γ^-/-^ mice against each individual protein of tetra-antigen TMV vaccine. **(A)** The wild-type (B6.IFN-γ^+/+^) and IFN-γ knockout (B6.IFN-γ^-/-^) mice were immunized i.n. on days 0 and 28 with the tetra-antigen TMV vaccine. **(B)** Antigen-specific total IgG antibody titers in immunized B6.IFN-γ^+/+^ and B6.IFN-γ^-/-^ mice against each individual protein of the tetra-antigen TMV vaccine were determined by ELISA on day 49 post-primary immunization. Pooled serum samples from immunized and the indicated control mice (*n* = 3 mice/group) were assayed in duplicate. The results are expressed as absorbance at 450 nm (mean ± standard deviation). The cut-off absorbance values used to determine the end-point titers are indicated by dashed lines and the actual values are shown in the box (top panels). The end-point titers are represented as Log_10_ values (bottom panel).

We also investigated the requirements for a protective immune response in mice vaccinated with tetra-antigen TMV vaccine. We immunized IFN-γ knockout (B6.IFN-γ^-/-^) mice with tetra-antigen TMV vaccine followed by a single booster immunization on day 28 (**Figure 9A**). The immunized mice were challenged i.n. with 10LD_100_ of *F. tularensis* LVS. It was observed that vaccinated or unvaccinated B6.IFN-γ^-/-^ mice died in a similar fashion and much earlier (median survival time, MST = 6) than the unvaccinated B6.IFN-γ^+/+^ control mice (MST = 8) (**Figure 9C**). These results indicate that IFN-γ is absolutely required for generation of a protective immune response in mice immunized with tetra-antigen TMV vaccine. The antibody responses in B6.IFN-γ^+/+^ and B6.IFN-γ^-/-^ mice immunized with tetra-antigen TMV vaccine on day 49 post-primary immunization were determined next. Analysis of *F. tularensis*-specific total IgG, IgG1, IgG2b, and IgG2c isotypes revealed no differences in antibody levels between immunized B6.IFN-γ^+/+^ and B6.IFN-γ^-/-^ mice (**Table 3**). Similarly, no differences in antigen-specific antibody responses were observed between immunized B6.IFN-γ^+/+^ and B6.IFN-γ^-/-^ mice (**Figures 10A,B**). These results demonstrate that the loss of protection in immunized B6.IFN-γ^-/-^ mice is not due to the lack of antibody production. However, this may probably be due to complete lack of a CMI response in immunized B6.IFN-γ^-/-^ mice.

**Table 3:** *F. tularensis-*specific antibody responses in B6.IFN-γ^+/+^ and B6.IFN-γ^-/-^ mice immunized with tetra-antigen TMV vaccine on day 49 post-primary immunization.

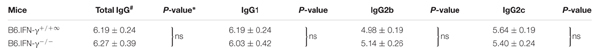


***^#^**Log_*10*_ titers expressed as mean ± SD (*n* = 3–4 mice/group). ^∞^These values are same as shown in **Table 1**. ^∗^*P*-values were determined by paired *t*-test. ns = not significant.*

## Discussion

Previous studies related to the development of tularemia subunit vaccines have provided evidence that a single antigenic component of *F. tularensis* is not sufficient for protection against a lethal respiratory challenge (Conlan et al., 2002; Apicella et al., 2010; Hickey et al., 2011; Kaur et al., 2012). On the other hand, studies using more than one purified antigenic components or a mixture of antigens either in the form of outer membrane proteins or *F. tularensis* LVS and SchuS4 lysates have shown improved protection against respiratory challenge with *F. tularensis* (Huntley et al., 2008; Ashtekar et al., 2012; Richard et al., 2014, 2017). These studies indicate that a complex mixture of antigens is needed in a vaccine formulation for the induction of a protective immune response. However, preparation of a complex mixture of antigens and their consistent delivery may pose a potential problem when a vaccine has to be manufactured on a large scale. In our previous study, we reported a novel approach to uniformly deliver multiple antigens of *F. tularensis* using TMV as a delivery platform. Using two different approaches for preparation of a multi-antigen TMV vaccine, we demonstrated that a cocktail of multiple TMV–protein conjugates rather than all the proteins conjugated to a single TMV virion is more effective in inducing a protective immune response (Banik et al., 2015). This approach provided a proof-of-concept for the efficacy of TMV as an efficient carrier and a suitable platform for delivery of multiple antigenic proteins of *F. tularensis*. There were several shortcomings in the vaccine formulation that was used. Specifically, we observed low protective efficacy of about 50% against a lethal respiratory challenge dose of *F. tularensis* LVS and to achieve even this level of protection, five immunizations by i.n. and s.c. routes were needed. The reason behind this low protective efficacy was that although the tri-antigen vaccine formulation consisting of DnaK, OmpA, and Tul4 proteins of *F. tularensis-*induced potent antigen-specific antibody responses, these were of T_H2_ type predominated by IgG1 antibodies with very low levels of IgG2a, IgG2b, and IgG2c isotype of antibodies were observed (Banik et al., 2015). This study, however, provided us with an excellent starting point for further vaccine improvement. In the current study, we refined the TMV vaccine formulation and vaccination strategy to reduce the number of immunizations, improve the level of protection to 100% against a lethal respiratory challenge with *F. tularensis* LVS, and to induce a potent and long-lasting protective immune response in immunized mice.

There are several advantages of using TMV as a carrier of multiple antigens of *F. tularensis*. Conjugation of antigens to TMV increases particle size thereby improves its immune uptake (Jennings and Bachmann, 2008; Manolova et al., 2008; Oyewumi et al., 2010). TMV has distinct advantages over nanoparticle and virus-like particle (VLP) formulations in that it is a naturally occurring virus, is highly uniform in structure, and can be easily produced at low cost. TMV provides a flexible backbone for association of any subunit protein antigen of interest from any source, and thus, can serve as a very versatile carrier for a multitude of antigens. TMV is stable for decades at room temperature, presenting the possibility that vaccine formulations with TMV may not require refrigeration. TMV is safe, as individuals world-wide are exposed to TMV in the food chain, and/or through handling tobacco products (Liu et al., 2013). Importantly, unlike other virus formulations, TMV does not get neutralized specifically after repeated boosting; either because of the limited immune reactivity of the TMV coat proteins, or because of the masking effect resulting from conjugation of proteins on its surface when used in a vaccine formulation (Mallajosyula et al., 2014). From an immunological stand point, the TMV architecture and size allow its rapid uptake by antigen presenting cells and the positive sense RNA core of TMV provides an additional adjuvant activity. Previous studies from our group have shown that a single dose immunization with TMV-hemagglutinin vaccine protects mice against influenza challenge (Mallajosyula et al., 2014). Moreover, a tri-antigen TMV vaccine protects 50% of the immunized mice against respiratory tularemia caused by *F. tularensis* LVS (Banik et al., 2015). Similarly, a recent study has demonstrated the suitability of TMV as a delivery platform for a subunit vaccine containing F1 and LcRV proteins of *Yersinia pestis* that provided complete protection against pneumonic plague in immunized mice (Arnaboldi et al., 2016). The goal of this study was to develop a fully protective tularemia subunit vaccine using TMV as a delivery platform for multiple antigens of *F. tularensis*.

We have developed a unique tetra-antigen vaccine formulation by incorporating an additional SucB protein into the tri-antigen formulation consisting of DnaK, OmpA, and Tul4 proteins of *F. tularensis*. The inclusion of these antigens was based on results from previous immunoproteomic studies and analysis of antibodies against these proteins in human subjects either infected naturally with Type A *F. tularensis* strains or vaccinated with LVS. Two of the four antigens, DnaK and SucB have almost exclusively been found to be reactive to serum from humans, non-human primates, mice, and rabbits immunized with *F. tularensis* LVS or from human patients who recovered from a known infection with *F. tularensis* SchuS4 (Havlasova et al., 2002, 2005; Eyles et al., 2007; Janovska et al., 2007). On the other hand, Tul4 has been shown to stimulate toll-like receptor 2 (TLR2) and elicit strong humoral as well as T-cell-mediated responses (Sjostedt et al., 1990, 1992; Valentino et al., 2009). Purified Tul4 from *F. tularensis* LVS incorporated into immunostimulating complexes when used as a vaccine reduced the hepatic and splenic colonization of *F. tularensis*, however, failed to induce any protection in immunized mice (Golovliov et al., 1995). An enhanced protective immune response was observed in immunized mice when recombinant Tul4 was used in combination with DnaK along with the GPI-0100 adjuvant (Ashtekar et al., 2012). Additionally, a combination of recombinant Tul4/FopA proteins when used as a vaccine prolonged the survival of immunized humanized mice following a challenge with *F. tularensis* LVS (Oh et al., 2017). The OmpA-like protein was also identified in immunoproteomic analysis (Huntley et al., 2007, 2008). Our previous studies have described the role of OmpA protein in immunopathogenesis and suppression of innate immune responses caused by *F. tularensis* (Mahawar et al., 2012; Dotson et al., 2013). These proteins were conjugated individually to the surface of TMV and then each protein–TMV conjugate was blended in equal concentrations to formulate a tetra-antigen TMV vaccine. As reported earlier (Eyles et al., 2007; Oh et al., 2016) to divert the IgG1-predominated T_H2_ response against the tri-antigen TMV vaccine, we included a CpG adjuvant; a TLR9 agonist and a potent inducer of T_H1_ type immune response in our vaccine formulation.

A successful conjugation of DnaK, OmpA, SucB, and Tul4 protein to TMV was observed by the disappearance of free protein and/or formation of high molecular weight complexes. The manner in which the subunit proteins conjugate with TMV depends on their molecular weights (Mallajosyula et al., 2014). The larger the subunit protein, the fewer molecules of subunit protein will associate with individual coat protein of the TMV (17 kD in size) due to steric hindrance at the virus surface (**Figure 1A**). This was specifically seen with DnaK protein and thus higher levels of free TMV coat proteins were observed at the end of conjugation reaction. On the other hand, other proteins may associate with TMV coat protein on the surface with least steric hindrance. Consequently, less amounts of free TMV coat proteins as indicated by less intense staining on the gels were observed at the end of successful conjugation with OmpA, SucB, and Tul4 proteins. The successful conjugation of these latter proteins to TMV resulted in formation of high molecular weight complexes that were too big to pass through the wells and thus were not seen on the gel (**Figure 1B**). In each of these conjugation reactions, we observed the presence of free TMV coat proteins depending on the conjugation efficacy of the protein with the TMV. We have observed that 100% of the control mice that receive TMV in equal concentration as the mice receiving TMV-conjugate vaccine succumb to infection following challenge with *F. tularensis* LVS (Banik et al., 2015). These observations indicate that the presence of free TMV in the tetra-antigen vaccine formulation may not contribute to any level of protection against the *F. tularensis* LVS challenge. On the other hand, the residual unconjugated proteins that may remain in our vaccine formulation may contribute to protection in parallel with the TMV-conjugated proteins. We observed that 50% of mice immunized on days 0 and 28 with a mixture of unconjugated recombinant DnaK, OmpA, SucB, and Tul4 proteins (10 μg each) along with the CpG (20 μg) adjuvant survived the lethal challenge with *F. tularensis* LVS (data not shown); while a similar vaccination regimen with TMV-conjugated proteins resulted in 80% survival (**Figure 9B**). However, it is worth noting that the concentrations of residual unconjugated proteins in our tetra-antigen TMV vaccine formulation are expected to be much lower than the mixture of unconjugated recombinant proteins used for immunization, and thus are not expected to contribute in a significant way to the protection observed in mice immunized with the tetra-antigen TMV vaccine. Previous studies using an identical approach for conjugation of antigenic proteins of influenza virus and *Y. pestis* to TMV have recorded 100% protection in mice against challenges with H1N1 influenza virus and CO92pgm strain of *Y. pestis*, respectively. However, the protection levels against influenza and *Y. pestis* were reduced to 20% and 50%, respectively, when unconjugated rather than TMV-conjugated proteins were used for immunizations (Arnaboldi et al., 2016). Collectively, these and the results from our studies demonstrate that conjugation of antigens to TMV enhances the protective immune response than those observed for a mixture of unconjugated proteins administered with or without adjuvants.

We demonstrate that immunizing mice with two booster immunizations on days 14 and 28 of the primary i.n. immunization with refined tetra-antigen TMV vaccine formulation provides 100% against a respiratory challenge with 10LD_100_ of *F. tularensis* LVS. Any alteration in this immunization regimen either by giving none or just one booster immunization reduces the protective efficacy of the vaccine. These results strongly suggest that the composition of the tetra-antigen vaccine that includes SucB has significantly improved protective efficacy even after just one dose. This is in contrast with previous observations that a prime-boost immunization strategy was absolutely required for achieving protection in mice immunized with subunit vaccines of *F. tularensis* (Jia et al., 2013; Richard et al., 2017; Roberts et al., 2017). The effectiveness of the tetra-antigen vaccine is evidenced by a sterilizing immunity, minimal pathology, potent antibody-mediated responses associated with a long-lasting recall, and prolonged duration of immunity. In contrast to the IgG1-predominated response observed in mice immunized with the tri-antigen vaccine without an adjuvant, the tetra-antigen vaccine induced mixed T_H1_ and T_H2_ biased antibody responses with higher levels of IgG2b and IgG2c antibodies. It has been reported that a mixed T_H1_ and T_H2_ response is required for protection against *F. tularensis* infection (Bitsaktsis et al., 2009). The inclusion of CpG adjuvant, which is a known TLR9 agonist with the capacity to activate innate immune cells, may have resulted in a switch toward a T_H1_ biased immunity associated with an increase in IgG2b and IgG2c levels.

Induction of a select group of cytokines such as IFN-γ and IL-17 in an *ex vivo* assay with splenocytes from immunized mice has been reported to be an indicator of protective efficacy and ability of the vaccine to induce a memory recall response (Paranavitana et al., 2010; De Pascalis et al., 2012; Mahawar et al., 2013). The recall responses were evaluated on day 84 post-primary immunization. Similar recall responses were observed in *ex vivo* assays irrespective of whether these were conducted using *F. tularensis* LVS or *F. tularensis* SchuS4-infected BMDMs. Induction of IFN-γ upon recall stimulation is a hallmark of memory response against *F. tularensis*. Moreover, a similar recall response against SchuS4 as that observed against *F. tularensis* LVS suggests that the tetra-antigen vaccine may also be protective against a *F. tularensis* SchuS4 challenge in mice. Levels of IFN-γ and IL-17 have been shown to be associated with protection against *F. tularensis* SchuS4 challenge in vaccinated mice. It has also been reported that these cytokines are induced immediately following challenge with *F. tularensis* SchuS4 (Lin et al., 2009; Khader and Gopal, 2010). Further, the IL-17-dependent induction of IFN-γ is required for the control of *F. tularensis* infection (Paranavitana et al., 2010). Collectively, our *ex vivo* assays demonstrate the ability of the tetra-antigen TMV vaccine to induce a strong memory recall response. The ability of the tetra-antigen TMV vaccine is also evidenced by the fact that boosting of immunized mice with half the dose of vaccine on day 91 post-primary immunization enhanced the antibody response on day 163 compared to that observed for day 84 post-primary immunization. This enhanced immune response also resulted in 80% protection in immunized mice when challenged on day 163 post-primary immunization. These studies convincingly demonstrate the long-term protection induced by the tetra-antigen TMV vaccine.

Interferon-gamma plays an important role in clearance of *Francisella* infection (Polsinelli et al., 1994). IFN-γ stimulates alveolar macrophages and recruits other phagocytic cells especially neutrophils to exert its protective effect in response to *Francisella* infection (Steiner et al., 2017). It has been shown that IFN-γ is required for protection during both the primary and secondary respiratory infection caused by *F. tularensis* and neutralization of IFN-γ in vaccinated mice results in a drastic weight loss and increased bacterial burden (Roberts et al., 2014). IFN-γ has also been shown to be important during the priming and effector phase of *F. tularensis* infection (Elkins et al., 2010). Moreover, passive transfer of antibodies into naïve IFN-γ^-/-^ mice fails to provide any protection against subsequent challenge with *F. tularensis* (Rhinehart-Jones et al., 1994; Kirimanjeswara et al., 2007). Our results show that vaccination of B6.IFN-γ^-/-^ mice fails to induce any degree of protection against a lethal i.n. challenge. The immunized B6.IFN-γ^-/-^ mice died similarly to unvaccinated B6.IFN-γ^-/-^ control mice. However, the B6.IFN-γ^-/-^ mice had similar levels of antibodies as observed in the wild-type B6.IFN-γ^+/+^ mice which were protected. These results indicate that loss of IFN-γ does not impair generation of antibody-mediated response but may affect the generation of T-CMI responses. However, adoptive transfer of T-cells from immunized wild-type mice or passive transfer of hyperimmune serum (Kirimanjeswara et al., 2007) to naïve IFN-γ^-/-^ mice fails to provide protection against subsequent i.n. or i.p. challenge with *F. tularensis.* Collectively, these results suggest that the B6.IFN-γ^-/-^ mice are not defective in the generation of *F. tularensis-*specific antibody responses. However, they completely lack cell-mediated responses. In the absence of T-cell help, antibodies alone fail to clear infection in IFN-γ^-/-^ mice immunized with tetra-antigen TMV. Based on these observations, it can be speculated that both primed T cells as well as *F. tularensis-*specific antibodies may be required for protection in IFN-γ^-/-^ mice. Nevertheless, results from the present study highlight the importance of IFN-γ in generation of a protective immune response.

We demonstrate that the tetra-antigen vaccine consisting of DnaK, OmpA, SucB, and Tul4 conjugated to TMV and admixed with CpG exhibits a better protective efficacy than DanK, OmpA, and Tul4 tri-antigen vaccine without any adjuvant (Banik et al., 2015). The better protective efficacy of this vaccine could result from a number of factors. Firstly, we observed that the humoral immune response generated following tetra-antigen vaccine with CpG was a mix of T_H1_ and T_H2_ type rather than a IgG1-predominated T_H2_ type response observed with the tri-antigen vaccine (Banik et al., 2015). This observation is supported by elevated pre-challenge levels of *F. tularensis* LVS-specific total IgG, IgG1, IgG2b, and IgG2c antibodies in tetra-antigen vaccinated mice. Secondly, potent SucB-specific antibody responses similar to those observed for DnaK and Tul4 were also generated in mice vaccinated with tetra-antigen vaccine. Additionally, CpG adjuvant induces a rapid and significant humoral immune response (Ciabattini et al., 2016), attenuation of T_H2_ type response, and induction of T_H1_ phenotype associated with IFN-γ or IL-17 dominated lung inflammation (Mirotti et al., 2017). We speculate that elevated levels of IgG2b and IgG2c antibodies may have resulted from inclusion of CpG adjuvant in our vaccine formulation. A combination of Tul4 and the outer membrane protein FopA along with the CpG adjuvant has been reported to enhance *in vitro* and *in vivo* immune responses compared to those observed for proteins alone, demonstrating the immune-potentiating action of the CpG adjuvant in the context of *F. tularensis* antigens (Oh et al., 2016). Our additional studies have shown that splenocytes from vaccinated mice induced similar *ex vivo* recall response following stimulation with DnaK, SucB, and Tul4 and these responses were of higher magnitude that those observed for splenocytes stimulated with OmpA protein (data not shown). These results indicate that addition of SucB to the vaccine formulation enhances the level of protection to 100%. However, SucB may not be the sole determinant of enhanced protective efficacy of the tetra-antigen TMV vaccine as other antigens of the vaccine formulation and the CpG adjuvant also contributes to the generation of a protective immune response.

To conclude, this study represents a very important milestone in the development of a multi-antigen subunit vaccine for prevention of tularemia. This study provides a proof-of-concept that a fully protective vaccine can be developed by incorporating the right formulation of protective antigens, using TMV as an i.n. delivery platform. Although this vaccine was not tested for its protective efficacy against the virulent *F. tularensis* SchuS4 strain, the recall response studies using *F. tularensis* SchuS4 provides support for the hypothesis that this vaccine may be effective against respiratory tularemia caused by *F. tularensis* SchuS4 as well. Furthermore, the results presented establish a foundation for the development of an effective vaccine against *F. tularensis* SchuS4 if the current formulation is only partially protective. Using TMV platform with a flexible plug-n-play approach allows for incorporation of additional *F. tularensis* antigens until a vaccine formulation than can provide complete protection against the fatal tularemia caused by *F. tularensis* SchuS4 is developed. Our future efforts are directed at developing a fully protective vaccine against *F. tularensis* SchuS4 using TMV-based delivery of multiple antigens of *F. tularensis* as described in this manuscript.

## Ethics Statement

The use of animals and protocols were approved by the Institutional Animal Care and Use Committee (IACUC) of New York Medical College (Protocol Number 22-2-0416H). This study was carried out in strict accordance with the recommendations and guidelines of National Council for Research (NCR) for care and use of animals. All the animal experiments were conducted in the centralized Animal Resources Facilities of New York Medical College licensed by the USDA and the NYS Department of Health, Division of Laboratories and Research, and accredited by the American Association for the Accreditation of Laboratory Care. Mice were administered an anesthetic cocktail consisting of ketamine (5 mg/kg) and xylazine (4 mg/kg) and underwent experimental manipulation only after they failed to exhibit a toe pinch reflex. Mice exhibiting more than 25% weight loss, anorexia, dehydration, and impairment of mobility were removed from the study and euthanized by approved means. Humane endpoints were also necessary for mice which survived at the conclusion of the experiment. Mice were administered an anesthetic cocktail of ketamine and xylazine intraperitoneally and then euthanized via cervical dislocation followed by cardiac puncture, a method that is consistent with recommendations of the Panel on Euthanasia of the American Veterinary Medical Association. In all experimental procedures, efforts were made to minimize pain and suffering.

## Author Contributions

MM, AMc, and CB conceived and designed the experiments. AMa, SB, RS, HK, MM, AMc, and CB performed the experiments. AMa, SB, MM, AMc, and CB analyzed the data. AMa, MM, and CB contributed reagents, materials, and analysis tools. AMc and CB wrote the paper.

## Conflict of Interest Statement

The authors declare that the research was conducted in the absence of any commercial or financial relationships that could be construed as a potential conflict of interest.

## Abbreviations

ATCC: American Type Culture Collection
BSL-3: biosafety level 3
CFU: colony forming units
CMI: cell-mediated immunity
EDC: 1-ethyl-3-(3-dimethylaminopropyl) carbodiimide hydrochloride
FDA: Food and Drug Administration
i.n.: intranasal
LD: lethal dose
LPS: lipopolysaccharide
LVS: live vaccine strain
MH: Mueller–Hinton
NHS: 5 mM *N*-hydroxysulfosuccinimide
TMV: tobacco mosaic virus
